# Lenvatinib in the Treatment of Differentiated Thyroid Cancer and Advanced Renal Cell Carcinoma

**Published:** 2017-11-01

**Authors:** Urvi J. Patel, Megan May

**Affiliations:** Emory University Hospital, Atlanta, Georgia, and Baptist Health Lexington Lexington, Kentucky

Differentiated thyroid cancer (DTC) is a malignancy that is occurring with greater frequency. Between the years 2006 and 2010, the incidence increased at an annual rate of 5.4% in men and 6.5% in women (Siegel, Ma, Zou, & Jemal, 2014). Although the prognosis of this cancer is favorable, optimal outcomes are only achieved through a multidimensional approach, which includes initial surgery to remove the thyroid and surrounding lymph nodes, followed by adjuvant therapy with radioactive iodine (RAI). 

Radioactive iodine is generally considered in patients with a high risk of locoregional recurrence or metastatic disease (which represents less than 10% of patients with clinical disease). This has been the mainstay of treatment for extrathyroidal disease, but it has been determined that two-thirds of these patients do not respond to RAI (RAI-refractory DTC; Durante et al., 2006; Trunnell & Brayer, 1953). Patients who develop RAI-refractory DTC have a 10-year overall survival rate of 19%, mainly due to the unresectable nature of the disease, which leads to disease progression.

Until 2013, the only US Food and Drug Administration (FDA)-approved treatment option for these patients was doxorubicin, but the treatment landscape is changing with the development of targeted therapies. The first targeted agent to be approved for the treatment of RAI-refractory DTC was sorafenib (Nexavar; Shah, Clayman, & Wirth, 2015). 

Renal cell carcinoma (RCC) accounts for nearly 2.6% of all cancers in men and women (Jemal et al., 2005). Primary treatment for localized RCC includes radical nephrectomy, involving the surgical removal of the kidney, the ipsilateral adrenal gland, and lymph nodes. Adjuvant therapy would be preferred in patients who are at high risk for locally advanced disease, but chemotherapy and immunomodulatory agents do not provide effective responses, resulting in poor prognosis. (Chemotherapy alone produces a 4%–6% rate of response in patients with advanced RCC [Cohen & McGovern, 2005].) Given the limited response rate of traditional therapies, an aggressive search for new therapies to abate the condition has ensued. The findings of this search have led to targeted therapies (Cohen & McGovern, 2005). 

Novel targeted therapies allow for an opportunity to significantly improve the treatment options of difficult-to-treat cancers such as DTC and RCC. Lenvatinib (Lenvima) is an oral multiple receptor tyrosine kinase inhibitor initially approved in February 2015 for the treatment of DTC that is recurrent, progressive, or refractory to RAI (FDA, 2015). As of May 2016, the agent has also been approved, in combination with everolimus (Afinitor), for the treatment of patients with advanced RCC who were previously treated with antiangiogenic therapy (FDA, 2016).

## PHARMACOLOGY/MECHANISM OF ACTION

Lenvatinib is a multitargeted tyrosine kinase inhibitor of vascular endothelial growth factor (VEGF) receptors VEGFR1 (FLT1), VEGFR2 (KDR), and VEGFR3 (FLT4); fibroblast growth factor receptors FGFR 1, 2, 3, and 4; as well as platelet-derived growth factor receptor alpha, KIT, and RET. Each of these receptor tyrosine kinases plays a key role in angiogenesis, which is one of the main processes that allows tumor growth and progress. Inhibition of these receptors allows for the minimization of an essential factor that the tumor needs to grow, allowing for a decrease in the tumor size and slowing of the progression of the cancer (Lexicomp Online, 2017a). 

When lenvatinib is used in combination with everolimus, which is a mechanistic target of rapamycin (mTOR) inhibitor, the antiangiogenic and antitumor activity of the drug is enhanced. This further minimizes the endothelial cell proliferation, tubule formation, and VEGF signaling that is an essential inhibitory mechanism necessary to combat RCC (Lexicomp Online, 2017a).

Lenvatinib is primarily metabolized by the hepatic enzyme CYP3A4 and aldehyde oxidase. Its half-life nears 28 hours, and it is mostly excreted through feces. The drug’s peak effect initiates 1 to 4 hours after consumption (Lexicomp Online, 2017a).

## CLINICAL TRIALS

**Differentiated Thyroid Cancer**

The SELECT trial was a multicenter, 2:1 randomized, double-blind, placebo-controlled phase III study that evaluated lenvatinib for the treatment of recurrent, progressive and/or refractory DTC. In this study, 392 patients with locally recurrent or metastatic RAI-refractory DTC who presented with radiographic evidence of disease progression within the 12 months prior to the initiation of the trial were randomized in a 2:1 ratio to treatment and placebo groups. The treatment group received 24 mg of lenvatinib once daily (Schlumberger et al., 2015). 

The major efficacy outcome measured was progression-free survival (PFS) using the Response Evaluation Criteria in Solid Tumors (RECIST). Secondary endpoints included objective response rate (ORR) and overall survival (OS). The study revealed the lenvatinib arm provided a statistically significant improvement in PFS (18.3 months, hazard ratio [HR], 0.21; 95% confidence interval [CI] = 0.16–0.28; *p* < .001) in comparison to the placebo arm (3.6 months). The lenvatinib arm also produced a statistically significant ORR (65%, *p* < .001) in comparison to the placebo arm (2%). Median OS had not been achieved in either treatment arm at the time of data collection (Schlumberger et al., 2015).

When the investigators focused on the subgroup of patients who had previously been treated with VEGF inhibitors (25% of the patients enrolled in the study), lenvatinib yielded significantly improved PFS (median, 15.1 months vs. 3.6 months in the placebo arm; HR, 0.22; 95% CI = 0.12–0.41; Schlumberger et al., 2015). These results show that lenvatinib has efficacy in the first-line and second-line settings for metastatic RAI-refractory DTC (Schlumberger et al., 2015).

**Renal Cell Carcinoma**

Lenvatinib, in combination with everolimus, was approved by the FDA for the treatment of advanced RCC following one prior antiangiogenic therapy after the completion of a phase II study. The combination therapy was granted Breakthrough Therapy designation, as preliminary evidence suggested substantial improvements over existing therapies in at least one endpoint as well as the combination’s intent to treat a serious or life-threatening disease (FDA, 2016).

The lenvatinib and everolimus combination is being studied in an ongoing 1:1:1 randomized, phase II multicenter, open-label study (ClinicalTrials.gov, 2017) across five countries. This study aims to determine the PFS among the three arms of the study: lenvatinib alone (24 mg once daily), lenvatinib plus everolimus (18 mg once daily and 5 mg once daily, respectively), and everolimus alone (10 mg once daily). Each treatment was administered continuously in 28-day cycles until disease progression or unacceptable toxicity developed (Motzer et al., 2015).

Publication of the initial findings from March 2012 to June 2013 indicated that 153 patients were randomized into the lenvatinib (n = 52), lenvatinib plus everolimus (n = 51), and everolimus (n = 50) groups. The study found that lenvatinib plus everolimus significantly improved PFS (14.6 months) when compared against everolimus alone (5.5 months, *p* = .0005) but not against lenvatinib alone (7.4 months, *p* = .12). The study also found that single-agent lenvatinib significantly prolonged PFS in comparison to everolimus alone (HR, 0.61; 95% CI = 0.38–0.98; *p* = .048). The lenvatinib plus everolimus arm also produced a statistically significant ORR (43%, *p* < .0001) in comparison to the single-agent everolimus arm (4%) but only a numerically improved ORR in comparison to the lenvatinib-alone group (27%; *p* = .10; Motzer et al., 2015).

An independent radiologic review was conducted to determine whether the efficacy results from the initial investigator assessment were reported accurately. This independent review supported the PFS benefits of lenvatinib plus everolimus in comparison to everolimus alone for patients with metastatic RCC whose disease progressed after one previous VEGF-targeted treatment (Motzer, Hutson, Ren, Dutcus, & Larkin, 2016). 

Although the data indicate both the single-agent lenvatinib and combination lenvatinib therapies provide an adequate response, the length of the ORR suggests the combination may prove to be more efficacious (Motzer et al., 2015).

## ADVERSE EFFECTS

The primary adverse effects of any grade that occurred in more than 40% of patients in the phase III SELECT trial in the lenvatinib group were hypertension (67.8%), diarrhea (59.4%), fatigue or asthenia (59.0%), decreased appetite (50.2%), decreased weight (46.4%), and nausea (41.0%; see Table 1 for additional adverse events). Discontinuation of the study drug due to adverse effects occurred in 37 patients who received lenvatinib (14.2%) and 3 patients who received placebo (2.3%). In the lenvatinib group, 6 of 20 deaths that occurred during the treatment period were considered to be drug-related (Schlumberger et al., 2015).

**Table 1:**
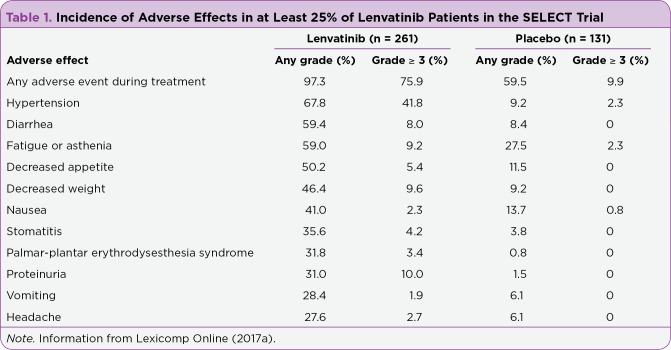
Incidence of Adverse Effects in at Least 25% of Lenvatinib Patients in the SELECT Trial

## CURRENT PLACE IN THERAPY

The FDA approval of lenvatinib supports its use as monotherapy in metastatic, progressive, or RAI-refractory DTC and combination therapy with everolimus in RCC that has been previously treated with an antiangiogenic agent. Progression-free survival associated with these regimens has proved to be significantly greater than that of its comparators (FDA, 2015, 2016). 

Several VEGF inhibitors have been studied in the treatment of both DTC and RCC as first- and second-line therapies. The trials looking at these agents include efficacy, comparative, and placebo-controlled studies. Each of these studies has demonstrated that targeted therapies provide beneficial antitumor activity in both cancers, especially in the second-line setting, where effective alternative systemic therapies have not been identified (Patel, Chaganti, & Motzer, 2006; Shah et al., 2015).

The National Comprehensive Cancer Network (NCCN) Guidelines for thyroid carcinoma support the use of lenvatinib in the treatment of metastatic thyroid cancer that is structurally persistent, refractory to RAI, and/or recurrent. The guidelines also state that other agents such as sorafenib can be used as alternative agents. Of the two agents, lenvatinib is the preferred drug, but the agent ultimately used is dependent on patient-specific factors such as comorbidities. These therapies are listed as an option, meaning they are based upon a lower level of evidence, but there is uniform consensus that the therapy is appropriate among the panelists who developed the guidelines. Other small-molecule kinase inhibitors not currently FDA approved for this indication can be considered if no clinical trials are available (NCCN, 2016). The monthly cost differential for the FDA-indicated regimens can be seen in Table 2.

**Table 2:**
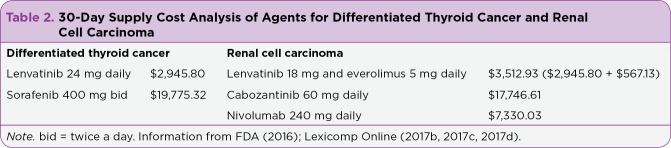
30-Day Supply Cost Analysis of Agents for Differentiated Thyroid Cancer and Renal Cell Carcinoma

The NCCN Guidelines for RCC state that the combination of lenvatinib plus everolimus is an option for the second-line treatment of metastatic RCC. This recommendation is based upon high-level evidence and a uniform consensus from the panelists who developed the guidelines. Although this is a strong recommendation, phase III studies (METEOR and CheckMate 025) state that eligible patients should receive cabozantinib (Cabometyx) and nivolumab (Opdivo) over everolimus. In these trials, cabozantinib and nivolumab were only studied against everolimus as monotherapy, so their preference over the combination of lenvatinib and everolimus cannot be accurately extrapolated (NCCN, 2017). The monthly cost differential for these regimens can be seen in Table 2.

## IMPLICATIONS FOR THE ADVANCED PRACTITIONER

Lenvatinib is a once-daily oral capsule used for DTC that is progressive, metastatic, or refractory to RAI and for RCC that has previously been treated with an antiangiogenic agent. This is an agent likely to be actively utilized by prescribers due to the lack of substantial treatment options for the previously mentioned indications as well as the development of resistance to alternative treatments within the same class (Lexicomp Online, 2017a).

The recommended dosing of lenvatinib is indication-based: 24 mg (two 10-mg capsules and one 4-mg capsule) for patients with DTC and 18 mg (one 10-mg capsule and two 4-mg capsules) for patients with RCC. Lenvatinib is dispensed in 30-day therapy packs. The 24-mg therapy pack comes with 90 capsules: sixty 10-mg capsules and thirty 4-mg capsules. The 18-mg therapy pack also comes with 90 capsules: thirty 10-mg capsules and sixty 4-mg capsules.

Regardless of indication, the medication is to be taken at the same time once a day without regard to food. The capsules should be swallowed whole, but if the patient is unable to do so, the capsules can be dissolved in a small amount of liquid. A missed dose should not be taken within 12 hours of the next dose, and the patient should never take two doses at one time. It is important to hydrate well throughout therapy (Lexicomp Online, 2017a).

Dosing adjustments for lenvatinib are needed for preexisting renal and hepatic impairment. If the patient presents with a creatinine clearance of less than 30 mL/min, the dose must be adjusted to 14 mg once daily for DTC and 10 mg once daily for RCC. If the patient suffers severe hepatic impairment (presents with Child-Pugh class C disease), the dose must be adjusted to 14 mg once daily for DTC and 10 mg once daily for RCC (Lexicomp Online, 2017a).

Major interactions associated with lenvatinib include the worsening of QTc prolongation when administered with other moderate- or high-risk QTc-prolonging agents. The risk associated with QTc-prolonging agents is stratified by the following categories: X (avoid combination), D (consider therapy modification), and C (monitor therapy). If combinations of lenvatinib and these drugs are used, electrocardiograms should be obtained to monitor for the prolongation of QTc. A complete list of agents and their risk stratification is provided in Table 3 (Lexicomp Online, 2017a).

**Table 3:**
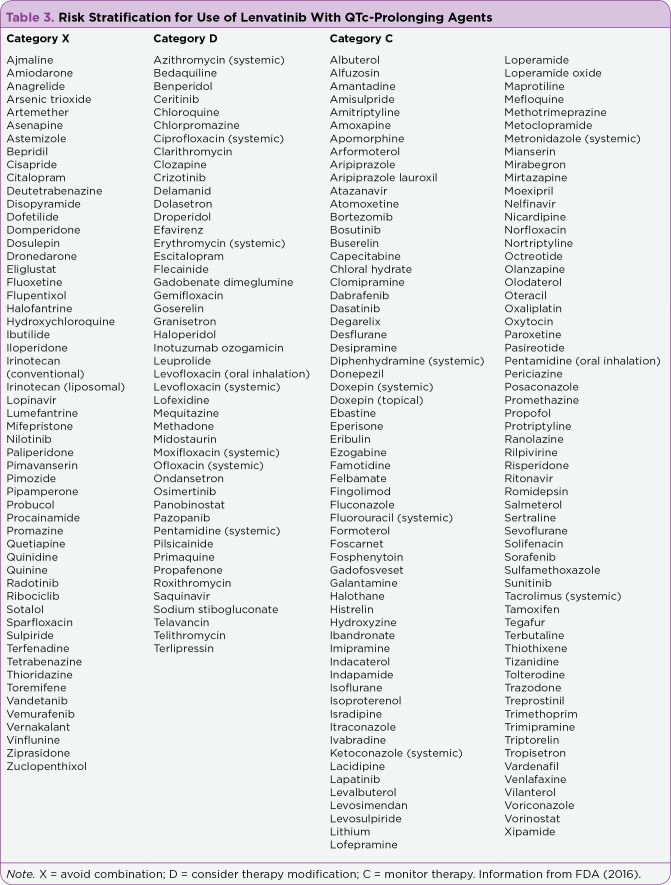
Risk Stratification for Use of Lenvatinib With QTc-Prolonging Agents

As lenvatinib is a UGT1A1 inhibitor, it can increase serum concentrations of irinotecan products, which are active metabolites of UGT1A1. The combination of lenvatinib and irinotecan products should be avoided. Although lenvatinib is primarily metabolized through CYP3A4, it is a minor substrate of the enzyme, so no dose adjustments or drug interactions need to be monitored (Lexicomp Online, 2017a).

In the DTC study, 68% of patients taking lenvatinib suffered adverse drug reactions (ADRs) that led to dose reduction (average 17.2 mg), and 18% had ADRs that led to treatment discontinuation. The primary reactions that led to dose reduction included hypertension and proteinuria (Cohen & McGovern, 2005). Dose interruptions and modifications are called for when persistent or intolerable grade 2 or 3 toxicities or grade 4 lab abnormalities are present. Dose interruptions should occur until the patient has recovered from the toxicity or it is minimized to a grade 1 or lower. The dose modifications occur based on the frequency of the ADR. The dose will be limited to 20 mg daily for a first-time ADR, 14 mg daily for the second time, and 10 mg daily for the third time. Table 4 provides a breakdown of the dose adjustments discussed here (Lexicomp Online, 2017a).

**Table 4:**
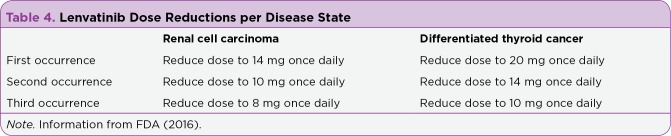
Lenvatinib Dose Reductions per Disease State

In the RCC study, 49% of patients taking lenvatinib and everolimus suffered ADRs that led to dose reduction, and 24% had ADRs that led to treatment discontinuation. The most common grade 3 and 4 toxicities associated with the combination regimen included diarrhea and hypertension (FDA, 2015). Dose interruptions and modifications are called for when persistent or intolerable grade 2 or 3 toxicities or grade 4 lab abnormalities are present. Dose interruptions should occur until the patient is recovered or the toxicity is minimized to a grade 1 or lower. The dose modifications should occur based on the frequency of the ADR. The dose will be limited to 14 mg daily for a first-time ADR, 10 mg daily for the second time, and 8 mg daily for the third time. Table 4 provides a breakdown of the dose adjustments discussed here (Lexicomp Online, 2017a).

For patients who have or develop hypertension, it is imperative to monitor blood pressure prior to and throughout the treatment course. Antihypertensive medications should be adjusted and maximized to achieve blood pressure control. If the patient’s hypertension is not controlled with a maximal antihypertensive regimen, lenvatinib therapy should be held until hypertension is ≤ grade 2.

For patients who develop ≥ 2 g proteinuria in 24 hours, lenvatinib therapy should be held. Proteinuria can be associated with an increase in blood pressure and an early sign of kidney damage. A dipstick test should be used to monitor these patients before initiation and periodically throughout treatment. If the urine dipstick proteinuria is ≥ 2+, then the patient needs a 24-hour urine protein level. Once the condition has resolved to < 2 g proteinuria in 24 hours, lenvatinib can be resumed at a reduced dose.

The suggested dose reductions are specific to the disease state being treated. For patients who develop diarrhea, prompt medical management is suggested prior to interrupting lenvatinib therapy. Once the diarrhea has resolved, lenvatinib may be reinitiated at the current or reduced dose. If diarrhea persists regardless of optimal medication management, lenvatinib therapy should be discontinued and not resumed. Disease-specific dose reductions can be found in Table 4 (Lexicomp Online, 2017a).

## SUMMARY

As seen in the SELECT study, lenvatinib alone can serve as primary and secondary options for the treatment of DTC refractory to RAI. The agent provides a positive PFS and durable response rate to a disease state with a previously poor prognosis. The currently ongoing trial evaluating lenvatinib use in advanced RCC shows that lenvatinib, alone or in combination with everolimus, provides greater PFS, but the length of the ORR indicates the combination regimen may be more beneficial. In either case, this agent offers a safer and more efficacious treatment alternative to patients who may not have had that opportunity previously. 

Clinical data discussed in this review have proved lenvatinib serves as an effective medication option in DTC and RCC, but there are other factors to consider prior to administering it. One such factor is the adverse-effect profile of lenvatinib. The SELECT and RCC studies reported the three primary adverse effects of lenvatinib that led to dose modifications were hypertension, proteinuria, and diarrhea. Two of these adverse effects (hypertension and diarrhea) can be medically managed prior to any dose reduction or discontinuation of therapy. Proteinuria, on the other hand, requires a hold in therapy followed by a dose reduction upon continuation. These side effects are comparable, and in some cases minimal, to the other agents that would be used for DTC and RCC.

Another factor to consider is the cost. Table 2 provides a breakdown of the average wholesale price associated with each lenvatinib regimen based on specific disease states. This table also compares lenvatinib with the FDA-approved therapeutic alternatives for each indication. In both DTC and RCC, the monthly cost of lenvatinib is lower than that of the other alternative regimens. 

Targeted therapies such as VEGF inhibitors have the potential to serve as first- and second- line treatment options for difficult-to-treat malignancies such as DTC and RCC. Their unique mechanism of action hinders a key process in tumor growth and survival, thus limiting disease progression and allowing for tumor shrinking. The response of these agents in patients whose malignancies have poor prognoses has garnered much excitement.
